# HIV Risk Profiles and Pre-Exposure Prophylaxis and Postexposure Prophylaxis Uptake Among Men Who Have Sex With Men in China: Cross-Sectional Latent Class Analysis

**DOI:** 10.2196/81405

**Published:** 2026-03-06

**Authors:** Ziwei Wu, Xinrui Zhang, Xingliang Zhang, Junfang Xu

**Affiliations:** 1 School of Public Health, Second Affiliated Hospital, School of Medicine, Zhejiang University, 866 Yuhangtang Road, Xihu District, Hangzhou, 310058, China, 86 18801230482; 2 Hangzhou Center for Disease Control and Prevention, Hangzhou Health Supervision Institution, Hangzhou, China

**Keywords:** men who have sex with men, postexposure prophylaxis, pre-exposure prophylaxis, PrEP or PEP uptake, latent class analysis, LCA, sexual behavioral factors, cognition of HIV preventive measures

## Abstract

**Background:**

Men who have sex with men remain disproportionately affected by HIV globally and in China. Despite the availability of pre-exposure prophylaxis (PrEP) and postexposure prophylaxis (PEP), their uptake remains suboptimal. Previous studies have rarely integrated both sexual behavioral factors and prevention-related cognitive factors. A clearer understanding of heterogeneity in HIV exposure and prevention literacy is needed to inform targeted HIV prevention strategies.

**Objective:**

This study aimed to identify latent subgroups of men who have sex with men based on sexual behaviors and prevention-related cognition and to examine differences in PrEP and PEP uptake across these subgroups.

**Methods:**

An online community–recruited cross-sectional survey was conducted among men who have sex with men in Hangzhou, China, from January 2024 to August 2024. A total of 3267 eligible participants (male at birth, aged ≥16 years, and history of sex with men) completed the structured self-administered questionnaires. Measures included sociodemographic characteristics, sexual behaviors within the past 3 months, exposure to HIV-related information, HIV testing history, and PrEP or PEP awareness and use. Latent class analysis was used to identify behavioral-cognitive subgroups. Multinomial logistic regression examined sociodemographic predictors of class membership, and binary logistic regression assessed associations between latent classes and PrEP or PEP use. A 2-sided *α* of .05 was used.

**Results:**

Four latent subgroups were identified: low-risk and well-informed (n=1219, 37.3%), high-risk and well-informed (n=735, 22.5%), moderate-risk and poorly informed (n=830, 25.4%), and high-risk and poorly informed (n=483, 14.8%). Overall, PrEP use was 7.81% (n=255; 95% CI 6.93%‐8.78%) and PEP use was 4.74% (n=155; 95% CI 4.07%‐5.53%). Compared with the low-risk, well-informed group, the high-risk, well-informed group had higher PrEP use (n=99, 13.48%; odds ratio [OR] 4.89, 95% CI 3.22‐7.42) and PEP use (n=69, 9.40%; OR 3.04, 95% CI 1.93‐4.77). The high-risk, poorly informed group also showed elevated PrEP use (n=65, 13.55%; OR 5.42, 95% CI 11.52%‐15.87%) and PEP use (n=31, 6.41%; OR 2.57, 95% CI 1.67‐3.98). The moderate-risk, poorly informed subgroup had the lowest uptake of PrEP (n=19, 2.30%; OR 0.89, 95% CI 1.35%‐3.90%) and PEP (n=9, 1.06%; OR 0.39, 95% CI 0.49%‐2.30%). Membership in poorly informed subgroups was associated with lower education, lower income, living in a rural area, and short-term residence.

**Conclusions:**

Distinct behavioral–cognitive typologies exist among men who have sex with men in Hangzhou, with substantial gaps between HIV risk exposure and prevention uptake. A large subgroup characterized by moderate behavioral risk but very low prevention literacy showed minimal PrEP or PEP use. These findings suggest that knowledge alone does not determine prevention utilization and highlight the need for integrated education, low-threshold counseling, and improved service access for underserved men who have sex with men. Linking behavioral–cognitive profiles to prevention outcomes provides actionable evidence for designing targeted and equitable HIV prevention strategies in real-world public health settings.

## Introduction

The Joint United Nations Program on HIV/AIDS reported that an estimated 39 million people were living with HIV by the end of 2023 in the whole world, with approximately 1.3 million new infections occurring in that year [1]. Although the overall global incidence has dropped by 59% since its peak in 1995, some key populations, especially men who have sex with men, continue to bear a disproportionate burden of new infections. For example, men who have sex with men accounted for an estimated 23% of new HIV infections in 2023 [1,2]. Notably, the proportion of new HIV infections attributed to men who have sex with men increased from approximately 11% in 2010 to 20% in 2022, reflecting a growing concentration of HIV transmission within this population [3]. In China, men who have sex with men accounted for 25.2% of newly reported HIV diagnoses nationwide, and in some cities, the proportion of newly notified HIV cases attributable to men who have sex with men approaches 50% in 2024 [4,5]. Such as, previous studies showed that the proportion of men who have sex with men among new HIV cases was 54.3% in Zhejiang, 74% in Hebei, and 76.6% in Tianjin [5]. Despite efforts including safer sex education, voluntary HIV counseling and testing, and peer-led interventions, the HIV epidemic persists among men who have sex with men in China [6]. These data highlight the urgent need to strengthen HIV prevention measures, with particular focus on the high-risk men who have sex with men groups.

Pre-exposure prophylaxis (PrEP) and postexposure prophylaxis (PEP) are proven to be effective ways to reduce the infection risk of HIV for men who have sex with men and are recommended by both World Health Organization (WHO) and the Chinese Center for Disease Control and Prevention [7,8]. While PEP has been available for occupational and nonoccupational exposure prevention since the late 1990s, PrEP represents a newer approach for ongoing protection before potential exposure. However, the uptake of PrEP and PEP remains limited in China, with only 20% of men who have sex with men aware of these options and fewer than 1% initiating PrEP since 2019 [9]. A multicity survey of men who have sex with men in 2021 reported that the usage rates remained low at 7.8% for PrEP and 9.5% for PEP, although with awareness rates of 65.9% and 78.7% respectively [10]. In China, PrEP has not yet been incorporated into the national health insurance system, and no formal subsidized program exists. Nevertheless, generic tenofovir disoproxil fumarate or emtricitabine formulations approved by the National Medical Products Administration in 2020 have lowered medication costs [9]. Men who have sex with men can access PrEP through infectious disease clinics, designated hospitals, and community-based programs in major cities, or seek information from local centers for disease control and prevention and online platforms, with professional consultation before initiation [11].

Empirical evidence indicates that sexual orientation and receptive versus insertive roles during anal intercourse contribute to differential biological vulnerability and network-mediated exposure to HIV [12,13]. Risk is further elevated by behaviors such as multiple sexual partnerships [14,15], group sex [16,17], and lack of awareness of partners’ HIV status [18,19]. Additionally, engagement in commercial sex, condomless anal intercourse, and substance use during sex are consistently associated with both increased HIV acquisition risk and higher likelihood of PrEP or PEP use [18,20-22]. A history of sexually transmitted infections (STIs) also serves as a clinical marker of biological susceptibility and behavioral risk clustering [23,24]. Cognitive and informational factors are equally critical in shaping prevention behavior. Exposure to HIV-related public health messaging enhances prevention literacy, while awareness of biomedical interventions (such as PrEP and PEP) and recent HIV testing experience are among the strongest predictors of uptake [23,25]. However, existing research has only examined sexual behavioral factors separately without accounting for the synergistic interactions or cumulative impacts of sexual behaviors as well as cognition of HIV preventive measures [12]. A more holistic evaluation of risk behaviors and prevention engagement is crucial for developing multidimensional vulnerability profiles among men who have sex with men.

In this context, we conducted a population-based cross-sectional study among men who have sex with men in China to examine associations between different HIV risk profiles (by considering both HIV-related risk behaviors and cognition of HIV preventive measures) and PrEP or PEP use. We aim to provide critical evidence for developing targeted HIV prevention interventions that address the diverse behavioral and cognitive profiles of men who have sex with men populations in China and comparable epidemiological settings.

## Methods

### Study Design and Participants

This study was designed, conducted, and reported in accordance with the STROBE (Strengthening the Reporting of Observational Studies in Epidemiology) guidelines for cross-sectional studies (Checklist 1) [26]. The survey was implemented in Hangzhou, Zhejiang Province, China. Participants were recruited via digital outreach of the Sunshine Coast Public Welfare Center (a community-based local nongovernmental organization [NGO] providing HIV-related education and services to men who have sex with men) between January 2024 and August 2024. The inclusion criteria for men who have sex with men were male at birth, aged 16 years or older, and reporting a history of anal or oral sex with a man. The link was disseminated on the NGO’s official WeChat account, community WeChat groups, and WeChat Mini Program, and the participants completed a structured, anonymous, self-administered questionnaire online. No a priori sample size calculation was performed because the study used community-based recruitment and aimed to characterize latent subgroups rather than estimate a single parameter.

### Data Measurement

#### Sample and Data Collection

The sociodemographic characteristics (eg, age, registered residence location and type, duration of residence in Hangzhou, marital status, employment status, educational attainment, and monthly income), sexual behavioral factors, cognition of HIV preventive measures, and experiences related to PrEP and PEP use were collected.

In our study, sexual behaviors over the preceding 3 months included sexual orientation (homosexual or bisexual), anal sex role (insertive, receptive, or versatile), number of male sex partners (0, 1, 2‐4, or ≥5), condom use, knowledge of partner’s HIV status, engagement in commercial sex (yes or no), unprotected sex (yes or no), drug use during intercourse (never or sometimes or every time), and any self-reported diagnosis of a STI within the past 3 months (yes or no). Knowledge of a partner’s HIV status was classified as “unknown” (participant did not know partner’s status), “partially known” (participant knew status of some but not all partners in the past 3 months, or partner reported but not documented), and “fully known” (participant reported that all recent partners’ HIV status was known, based on partner disclosure or test results). Self-reported STI diagnosis referred to any clinician-diagnosed STI within the past 3 months, including syphilis, gonorrhea, genital herpes, genital warts, chlamydia trachomatis, and other self-reported infections.

Cognition of HIV preventive measures included whether they had been exposed to HIV-related information, undergone HIV testing, and their awareness and uptake of biomedical HIV prevention strategies (ie, PrEP and PEP). Awareness of medication-based HIV prevention was measured using a single yes or no composite item: “Have you ever heard of HIV prevention medications such as PrEP or PEP?” This composite question was used to minimize respondent burden and to improve comprehension among participants with varying biomedical literacy. While some studies assess PrEP and PEP awareness separately, the combined phrasing has also been used in prior research and surveys assessing baseline chemoprophylaxis awareness [27]. PrEP and PEP uptake were assessed using 2 separate yes or no items: “Have you used PrEP in the past 3 months?” and “Have you used PEP in the past 3 months?” These outcomes were modeled using binary logistic regression, with latent sexual behavior class membership as the primary independent variable and sociodemographic characteristics as covariates.

Most sexual-behavior items were single-item behavioral self-reports (number of partners, condom use, and drug use during sex) rather than multi-item psychometric scales. For cognition or awareness items, we used direct single-item indicators (exposure to HIV publicity, awareness of PrEP, and awareness of PEP); these were face-valid and widely used in similar men who have sex with men surveys [28,29].

There were no missing data for variables included in the analysis because the online survey platform required all items to be answered before submission (forced-response design). Therefore, no imputation procedures were necessary. Variables of both sexual behavioral factors and cognition of HIV preventive measures were considered to identify latent risk profiles of men who have sex with men.

#### Bias

To enhance data quality and reduce potential sources of bias, several procedural safeguards were implemented during data collection. The survey was administered anonymously online without collecting any personal identifiers, which minimized interviewer influence and encouraged candid reporting. All items were mandatory within the survey system to ensure completeness of responses. A 6-month reference frame was used for behavioral questions to improve accuracy and consistency of recall. Recruitment was conducted broadly through a major men who have sex with men community-based organization to maximize reach within the target population. These methodological steps were designed to strengthen internal validity and reduce measurement error.

### Ethical Considerations

The study protocol and consent procedure were approved by the Medical Ethics Committee of the School of Public Health, Zhejiang University (project ZGL202306-9). Electronic informed consent was obtained at the beginning of the online survey; participants proceeded only after checking an “I agree” box. Survey responses were anonymous; no personal identifiers were collected. Data were stored on a password-protected server accessible only to study staff. No compensation was provided. No images containing identifiable participant information were included.

### Statistical Analyses

Descriptive statistics were used to describe the sociodemographic characteristics and sexual behavior profiles of the incorporated men who have sex with men participants. Latent class analysis (LCA) was conducted using the *poLCA* package (1.6.0.1) in R (version 4.3.3; R Foundation for Statistical Computing) to identify distinct behavioral and cognitive subgroups based on the main factors influencing the use of PrEP and PEP (ie, sexual behaviors and cognition of HIV preventive measures). LCA models with 2 to 5 latent classes were estimated, and model fit was assessed using multiple indices, including the Akaike information criterion (AIC), Bayesian information criterion (BIC), adjusted BIC (aBIC), entropy, Vuong-Lo-Mendell-Rubin likelihood ratio test (VLMR-LRT), bootstrapped likelihood ratio test (BLRT) [30], and average latent class posterior probability (ALCPP) [31]. Lower AIC, BIC, and aBIC values, and higher entropy values, indicate better model fit. Entropy values of more than 0.60 were considered indicative of acceptable model fit [32], while ALCPP values of more than 0.70 indicated reliable classification [31]. The VLMR-LRT and BLRT were used to compare the relative fit of K-class versus (K–1)-class models. Prior research suggests that among fit indices, BLRT exhibits the highest performance, followed by BIC and aBIC [33]. Additionally, selection of the optimal model also considered the interpretability, meaningfulness, and proportional size of latent classes [34]. In case of conflicting fit indices, we prioritized BLRT and BIC or aBIC for statistical fit, and then considered entropy, ALCPP, minimum class size (>5% a priori), and interpretability.

Following the identification of latent sexual behavior subgroups via LCA, further analyses were conducted to examine differences across classes. Multinomial logistic regression models were used to assess associations between sociodemographic variables and latent sexual behavior class membership. In addition, latent classes were used as the main independent variable in binary logistic regression models to explore their association with the PrEP and PEP uptake, while adjusting for the same set of sociodemographic covariates. All statistical analyses were conducted using SPSS (Statistical Package for Social Sciences; version 29.0; IBM Corp). A 2-sided *P* value <.05 was considered statistically significant. Results were reported as odds ratios (ORs) with corresponding 95% CIs.

## Results

### Characteristics of Men Who Have Sex with Men

The Sunshine Coast Public Welfare Center’s online platform serves approximately 10,700 men who have sex with men residing in Hangzhou. A total of 3267 participants were recruited (from January 2024 to August 2024), yielding an estimated response rate of 30.52%. The majority of participants were young, with 58.4% aged 16 to 29 years (Table 1). Most participants (n=2179, 66.7%) had resided in the city for more than 2 years. Educational attainment was generally high, with 46.8% (n=1529) holding a bachelor’s degree and 12.5% (n=407) attaining a master’s degree or higher. The income distribution suggested a moderate socioeconomic profile, with 45.1% (n=1473) earning between 5000 and 10,000 RMB (1 RMB=US $0.14) per month, and 29.2% (n=1043) reporting monthly earnings above 10,000 RMB.

**Table 1.** Sociodemographic and behavioral characteristics of men who have sex with men participants, Hangzhou, China, January 2024 to August 2024 (N=3267).

Items	Frequency, n (%)
Age (y)	
Approximately 16-29	1908 (58.4)
30‐49	1236 (37.8)
≥50	123 (3.8)
Duration of residence in Hangzhou	
<6 months	323 (9.9)
Approximately 6-12 months	257 (7.9)
1-2 years	477 (14.6)
>2 years	2179 (66.7)
Not in Hangzhou	31 (0.9)
Registered residence	
Zhejiang province	1496 (45.8)
Other provinces	1771 (54.2)
Type of registered residence	
Urban	1487 (45.5)
Rural	1780 (54.5)
Occupation	
Student	432 (13.2)
Full-time (public sector)	228 (7)
Full-time (nonpublic sector)	1865 (57.1)
Part-time	70(2.1)
Unemployed	177 (5.4)
Retired	21 (0.6)
Other	474 (14.5)
Marital status	
Single/never married	2696 (82.5)
Cohabiting	99 (3)
Divorced or widowed	96 (2.9)
Married	376 (11.5)
Education level	
Primary school or below	29 (0.9)
Junior high school	171 (5.2)
Senior high school	258 (7.9)
Vocational or technical school	119 (3.6)
Associate degree	754 (23.1)
Bachelor’s degree	1529 (46.8)
Master’s degree or higher	407 (12.5)
Monthly income (RMB^a^)	
<3000	415 (12.7)
3000-4999	424 (13)
5000-10,000	1473 (45.1)
>10,000	955 (29.2)
Sexual orientation	
Homosexual	2224 (68.1)
Bisexual	1043 (31.9)
Sexual role during sexual behaviors	
Receptive (0)	680 (20.8)
Versatile (0.5)	1529 (46.8)
Insertive (1)	1058 (32.4)
Sexual partners^b^	
0	745 (22.8)
1	1455 (44.5)
Approximately 2-4	939 (28.7)
≥5	128 (3.9)
Awareness of partners’ HIV status	
Not at all aware	494 (15.1)
Partially aware	1187 (36.3)
Fully aware	1586 (48.5)
Engaged in commercial sex^b^	
Yes	89 (2.7)
No	3178 (97.3)
Had unprotected sex^b^	
Yes	1044 (32)
No	2223 (68)
Used drugs during sex^b^	
Never	2462 (75.4)
Sometimes	649 (19.9)
Every time	156 (4.8)
Diagnosed with STI^b^^,^^c^	
Syphilis	34 (1)
Gonorrhea	2 (0.1)
Genital herpes	1 (0)
Genital warts (HPV^d^)	10 (0.3)
*Chlamydia trachomatis*	0 (0)
Other	19 (0.6)
None	3201 (98)
Saw HIV-related publicity^b^	
Yes	2496 (76.4)
No	771 (23.6)
Awareness of PrEP^e^ and PEP^f^	
Not at all aware	311 (9.5)
Partially aware	1791 (54.8)
Fully aware	1165 (35.7)
Used PrEP^b^	
Yes	255 (7.8)
No	3012 (92.2)
Used PEP^b^	
Yes	155 (4.7)
No	3112 (95.3)
Tested for HIV in the past 6 months	
Yes	2470 (75.6)
No	797 (24.4)

a 1 RMB=US $0.14.

b Within 3 months before this survey.

c STI: sexually transmitted infection.

d HPV: human papillomavirus.

e PrEP: pre-exposure prophylaxis.

f PEP: postexposure prophylaxis.

A considerable proportion (68.1%) self-identified as homosexual, whereas 31.9% reported being bisexual. Nearly half of the respondents (44.5%) reported having one male sexual partner in the past 3 months, while 28.7% had 2 to 4 partners, and 3.9% reported 5 or more. Risk-related sexual behaviors were prominent, with 32% engaging in unprotected intercourse and 2.7% reporting engagement in commercial sex. Drug use during sexual activity was reported by 4.8% of participants. Only 48.5% of respondents reported being fully informed of their partners’ status. Only 2% of men who have sex with men reported being diagnosed with STIs, with 1% with syphilis. Most participants (75.6%) had received HIV testing within the previous six months.

Despite high exposure to HIV-related education campaigns (76.4%), only 35.7% demonstrated awareness of PrEP and PEP, with only 7.8% having used PrEP and 4.7% having used PEP.

### Model Fitting and Selection

LCA was conducted to compare models comprising 2 to 5 latent classes (Table 2). The ALCPP declined from 0.892 in the 2-class model to 0.757 in the 5-class model, and entropy values were consistently low—particularly for the 3-class (0.591) and 5-class (0.619) models—indicating reduced classification precision with increasing numbers of classes. Both the VLMR-LRT and the BLRT yielded statistically significant results (*P*<.001), indicating improved model fit with the inclusion of additional classes. Specifically, the 2-class model exhibited the most distinct classification structure but demonstrated poor overall model fit, whereas the 3-class model showed improved fit but suffered from the lowest entropy value. The 4-class model provided the optimal tradeoff among model fit (as reflected by lower AIC and BIC values), entropy (0.630), and classification reliability (ALCPP=0.787), and was therefore selected as the final solution. The 5-class model demonstrated signs of overfitting and diminished clarity in latent class differentiation.

**Table 2.** Fit statistics for latent class solutions by number of classes.

Classes	AIC^a^	BIC^b^	aBIC^c^	Log-likelihood	ALCPP^d^	Entropy	Minimum sample size for the class (n)	VLMR-LRT^e^	BLRT^f^
2	49363.0	49637.1	49858.2	−24636.5	0.892	0.633	45	—^g^	—
3	48726.5	49140.7	49474.8	−24295.2	0.804	0.591	68	<.001	<.001
4	48496.4	49050.7	49497.9	−24157.2	0.787	0.630	91	<.001	<.001
5	48324.6	49019.1	49579.2	−24048.3	0.757	0.619	114	<.001	<.001

a AIC: Akaike information criteria.

b BIC: Bayesian information criteria.

c aBIC: adjusted Bayesian information criteria.

d ALCPP: average latent class posterior probability.

e VLMR-LRT: Vuong-Lo-Mendell-Rubin likelihood ratio test.

f BLRT: bootstrapped likelihood ratio test.

g Not applicable.

Accordingly, 4 distinct latent classes (ie, low-risk and well-informed class, high-risk and well-informed class, moderate-risk and poorly informed class, and high-risk and poorly informed class) were identified, characterized by varying sexual behaviors and cognition of HIV preventive measures (Table 3). *The low-risk, well-informed group* exhibited the most protective behavioral profile: only 2.4% reported having 2 or more partners in the past 3 months, no participants engaged in condomless sex, and 74.9% had undergone HIV testing within that period. Awareness of PrEP and PEP was moderate (42.3%), and a substantial proportion (87.4%) reported recent exposure to HIV-related public health messaging. *The high-risk, well-informed group* reported elevated sexual risk behaviors—38.1% had two or more partners and 67.2% engaged in condomless sex—yet demonstrated strong prevention engagement: 77.3% were fully aware of PrEP and PEP, 88.5% had undergone recent HIV testing, and 97% knew their partners’ HIV status. *The moderate-risk, poorly informed group* displayed intermediate levels of behavioral risk—24.1% reported multiple partners and 23.5% reported condomless sex—but exhibited the lowest levels of prevention knowledge: 99.8% lacked full awareness of PrEP or PEP, 80.4% had not encountered HIV-related messaging, and only 48.5% had recently undergone testing. *The high-risk, poorly informed group* represented the most vulnerable subgroup: 72% reported multiple partners, 55.9% engaged in condomless sex, and 76.3% lacked comprehensive awareness of PrEP and PEP. Despite a relatively high rate of HIV testing (85%), prevention literacy remained consistently low across this group.

**Table 3.** HIV risk-clustering characteristics of men who have sex with men participants, Hangzhou, China, January 2024 to August 2024 (N=3267).

Characteristic	Class 1	Class 2	Class 3	Class 4
Participants, n (%)	1186 (36.3)	564 (17.3)	565 (17.3)	952 (29.1)
95% CI	34.7-38.0	16.0-18.6	16.0-18.6	27.6-30.7
Sexual orientation, n (%)				
Homosexual	783 (66)	439 (77.8)	290 (51.3)	712 (74.8)
Bisexual	403 (34)	125 (22.2)	275 (48.7)	240 (25.2)
Sexual role during anal sex with a man, n (%)				
Receptive (0)	215 (18.1)	129 (22.9)	108 (19.1)	228 (23.9)
Versatile (0.5)	571 (48.1)	213 (37.8)	297 (52.6)	44 8(47.1)
Insertive (1)	400 (33.7)	222 (39.4)	160 (38.3)	276 (29)
Number of sexual partners, n (%)^a^				
0	527 (44.4)	42 (7.4)	162 (28.7)	14 (1.5)
1	631 (53.2)	307 (54.4)	267 (47.3)	250 (26.3)
Approximately 2-4	28 (2.4)	202 (35.8)	128 (22.7)	581 (61)
≥5	0 (0)	13 (2.3)	8 (1.4)	107 (11.2)
Awareness of partners’ HIV status, n (%)				
Not at all aware	124 (10.5)	17 (3)	216 (38.2)	137 (14.4)
Partially aware	298 (25.1)	0 (0)	203 (35.9)	686 (72.1)
Fully aware	764 (64.4)	547(97)	146 (25.8)	129 (13.6)
Engaged in commercial sex, n (%)^a^				
No	1178 (99.3)	519 (92)	558 (98.8)	923 (97)
Yes	8 (0.7)	45 (8)	7 (1.2)	29 (3)
Had unprotected sex, n (%)^a^				
No	1186 (100)	185 (32.8)	432 (76.5)	420 (44.1)
Yes	0 (0)	379 (67.2)	133 (23.5)	532 (55.9)
Used drugs during sex, n (%)^a^				
Never	1146 (96.6)	348 (61.7)	474 (83.9)	494 (51.9)
Sometimes	25 (2.1)	157 (27.8)	63 (11.2)	404 (42.4)
Every time	15 (1.3)	59 (10.5)	28 (5)	54 (5.7)
Diagnosed with STIs^b^, n (%)^a^				
None	1159 (97.7)	552 (97.9)	560 (99.1)	930 (97.7)
Syphilis	14 (1.2)	4 (0.7)	5 (0.9)	11 (1.2)
Gonorrhea	1 (0.1)	0 (0)	0 (0)	1 (0.1)
Genital warts (HPV^c^)	0 (0)	0 (0)	0 (0)	1 (0.1)
*Chlamydia trachomatis*	0 (0)	6 (1.1)	0 (0)	4 (0.4)
Other	12 (1)	2 (0.4)	0 (0)	5 (0.5)
Saw HIV-related publicity, n (%)^a^				
No	149 (12.6)	29 (5.1)	454 (80.4)	139 (14.6)
Yes	1037 (87.4)	535 (94.9)	111 (19.6)	813 (85.4)
Awareness of PrEP^d^ and PEP, n (%)^e^				
Not at all aware	0 (0)	9 (1.6)	297 (52.6)	5 (0.5)
Partially aware	684 (57.7)	119 (21.1)	267 (47.3)	721 (75.7)
Fully aware	502 (42.3)	436 (77.3)	1 (0.2)	226 (23.7)
Tested for HIV in the past 6 months, n (%)				
No	298 (25.1)	65 (11.5)	291 (51.5)	143 (15)
Yes	888 (74.9)	499 (88.5)	274 (48.5)	809 (85)

a Within 3 months before this survey.

b STI: sexually transmitted infection.

c HPV: human papillomavirus.

d PrEP: pre-exposure prophylaxis.

e PEP: postexposure prophylaxis.

### Composition of 4 HIV Risk-Related Behavior Patterns

As shown in Table 4, men who have sex with men aged 16 to 29 years had significantly lower odds of belonging to the moderate-risk, poorly informed class compared with those aged 50 years or older (OR 0.452, 95% CI 0.255-0.798; *P*=.006). Household registration characteristics were significantly associated with latent class membership. Participants with registered residence in Zhejiang province had lower odds of being in the moderate-risk, poorly informed class (OR 0.745, 95% CI 0.596-0.931; *P*=.01) and the high-risk, poorly informed class (OR 0.759, 95% CI 0.631-0.912; *P*=.003) compared with those from other provinces. Similarly, individuals from urban areas had reduced odds of being classified into the high-risk, poorly informed class compared with those from rural areas (OR 0.808, 95% CI 0.671-0.972; *P*=.02). Full-time employment in the public sector was associated with lower odds of membership in the moderate-risk, poorly informed class compared with other occupational categories (OR 0.559, 95% CI 0.317-0.987; *P*=.045). Educational attainment was strongly associated with class membership. Compared with those holding a master’s degree or above, participants with primary school or below (OR 3.496, 95% CI 1.268-9.639; *P*=0.02), junior high school (OR 2.482, 95% CI 1.380-4.463; *P*=0.002), senior high school (OR 2.279, 95% CI 1.379-3.766; *P*=0.001), vocational or technical education (OR 2.922, 95% CI 1.551-5.504; *P*<0.001), and associate degree (OR 1.880, 95% CI 1.253-2.822; *P*=0.002) had higher odds of belonging to the moderate-risk, poorly informed class. Monthly income was inversely associated with membership in the high-risk, poorly informed class. Compared with those earning more than 10,000 RMB per month, participants earning less than 3000 RMB (OR 0.620, 95% CI 0.416-0.923; *P*=.02), 3000 to 4999 RMB (OR 0.726, 95% CI 0.529-0.996; *P*=.047), and 5000 to 10,000 RMB (OR 0.682, 95% CI 0.550-0.847; *P*<.001) had lower odds of belonging to the high-risk, poorly informed class.

**Table 4.** Association between sociodemographic characteristics and HIV risk patterns of men who have sex with men participants, Hangzhou, China, January 2024 to August 2024 (N=3267)^a^.

Characteristics	High-risk, well-informed class		Moderate-risk, poorly informed class		High-risk, poorly informed class	
	OR^b^ (95% CI)	*P* value	OR (95% CI)	*P* value	OR (95% CI)	*P* value
Age (y)						
Approximately 16-29	1.686 (0.717-3.967)	.23	0.452 (0.255-0.798)	.006	1.854 (0.949-3.623)	.07
30‐49	1.261 (0.548-2.901)	.59	0.510 (0.302-0.862)	.01	1.756 (0.919-3.354)	.09
≥50	1.000		1.000		1.000	
Duration of residence in Hangzhou						
<6 months	2.002 (0.399-10.047)	.40	0.412 (0.148-1.149)	.09	0.577 (0.208-1.601)	.29
Approximately 6-12 months	1.686 (0.333-8.522)	.53	0.392 (0.139-1.105)	.08	0.469 (0.168-1.315)	.15
Approximately 1-2 years	1.672 (0.336-8.312)	.53	0.342 (0.124-0.939)	.04	0.488 (0.178-1.335)	.16
>2 years	1.648 (0.336-8.078)	.54	0.364 (0.137-0.973)	.04	0.545 (0.203-1.464)	.23
Not in Hangzhou	1.000		1.000		1.000	
Registered residence						
Zhejiang province	1.057 (0.853-1.309)	.61	0.745 (0.596-0.931)	.01	0.759 (0.631-0.912)	.003
Other provinces	1.000		1.000		1.000	
Type of registered residence						
Urban	1.119 (0.902-1.388)	.31	0.932 (0.746-1.164)	.54	0.808 (0.671-0.972)	.02
Rural	1.000		1.000		1.000	
Occupation						
Student	1.358 (0.853-2.162)	.20	0.986 (0.604-1.611)	.96	0.799 (0.525-1.215)	.29
Full-time (public sector)	1.449 (0.912-2.303)	.12	0.559 (0.317-0.987)	.045	1.069 (0.718-1.592)	.74
Full-time (non–public sector)	1.073 (0.775-1.486)	.67	1.169 (0.865-1.580)	.31	1.039 (0.801-1.348)	.77
Part-time	1.678 (0.785-3.587)	.18	1.282 (0.591-2.779)	.53	1.406 (0.731-2.704)	.31
Unemployed	1.003 (0.579-1.740)	.99	1.179 (0.700-1.984)	.54	1.118 (0.716-1.746)	.62
Retired	3.183 (0.611-16.567)	.17	1.385 (0.367-5.226)	.63	3.567 (0.928-13.707)	.06
Other	1.000		1.000		1.000	
Marital status						
Single or never married	1.362 (0.876-2.118)	.17	0.617 (0.431-0.882)	.008	1.029 (0.734-1.443)	.87
Cohabiting	1.942 (0.947-3.979)	.07	0.980 (0.491-1.956)	.95	1.513 (0.825-2.773)	.18
Divorced or widowed	0.652 (0.264-1.608)	.35	0.973 (0.542-1.749)	.93	1.087 (0.605-1.953)	.78
Married	1.000		1.000		1.000	
Education level						
Primary school or lower	2.969 (1.053-8.375)	.04	3.496 (1.268-9.639)	.02	0.204 (0.025-1.678)	.14
Junior high school	1.351 (0.655-2.786)	.42	2.482 (1.380-4.463)	.002	1.851 (1.093-3.133)	.02
Senior high school	1.651 (0.994-2.742)	.05	2.279 (1.379-3.766)	.001	1.246 (0.801-1.939)	.33
Vocational or technical school	2.186 (1.140-4.189)	.09	2.922 (1.551-5.504)	<.001	1.942 (1.109-3.401)	.02
Associate degree	1.501 (1.036-2.174)	.03	1.880 (1.253-2.822)	.002	1.344 (0.973-1.857)	.07
Bachelor’s degree	1.062 (0.773-1.460)	.71	1.050 (0.726-1.519)	.80	1.027 (0.777-1.357)	.85
Master’s degree or higher	1.000		1.000		1.000	
Monthly income (RMB^c^)						
<3000	0.720 (0.465-1.114)	.14	1.172 (0.741-1.855)	.50	0.620 (0.416-0.923)	.02
Approximately 3000-4999	0.562 (0.381-0.830)	.004	0.823 (0.560-1.210)	.32	0.726 (0.529-0.996)	.047
Approximately 5000-10,000	0.718 (0.556-0.927)	.01	0.902 (0.687-1.185)	.46	0.682 (0.550-0.847)	<.001
>10,000	1.000		1.000		1.000	

a The "low-risk, well-informed class" was included in the model as the reference group.

b OR: odds ratio.

c 1 RMB=US $0.14.

### Uptake of PEP and PrEP Across HIV Risk Patterns

Table 5 revealed substantial disparities in the uptake of PrEP and PEP across distinct HIV risk profiles. Overall, PrEP use was 7.81% (255/3267; 95% CI 6.93%-8.78%) and PEP use was 4.74% (155/3267; 95% CI 4.07%-5.53%). Compared with the low-risk, well-informed reference group (PrEP: 37/1186, 3.12%; PEP: 35/1186, 2.95%), individuals in the high-risk, well-informed class reported significantly higher use of PrEP (76/564, 13.48%; OR 4.890, 95% CI 3.224-7.418, *P*<.001) and PEP (53/564, 9.40%; OR 3.035, 95% CI 1.930-4.771, *P*<.001). Similarly, participants in the high-risk, poorly informed class also showed higher PrEP uptake (129/952, 13.55%; OR 5.419, 95% CI 3.690-7.959, *P*<.001) and higher PEP use (61/952, 6.41%; OR 2.574, 95% CI 1.666-3.978, *P*<.001) compared with the reference group. In contrast, the moderate-risk, poorly informed group showed no significant difference in PrEP use (13/565, 2.30%; OR 0.891, 95% CI 0.463-1.713, *P*=.73) and had significantly lower PEP uptake (6/565, 1.06%; OR 0.385, 95% CI 0.158-0.939, *P*=.04) relative to the reference group.

**Table 5.** Association between HIV risk patterns and the use of pre-exposure prophylaxis (PrEP) and postexposure prophylaxis (PEP) by men who have sex with men participants, Hangzhou, China, January 2024 to August 2024 (N=3267).

Class	PrEP			PEP		
	Used, n (%; 95% CI)	Odds ratio (95% CI)	*P* value	Used, n (%; 95% CI)	Odds ratio (95% CI)	*P* value
Overall	255 (7.81; 6.93-8.78)	—^c^	—	155 (4.74; 4.07-5.53)	—	—
Low-risk, well-informed class (n=1186)	37 (3.12; 2.27-4.27)	1.000	<.001	35 (2.95; 2.13-4.08)	1.000	<.001
High-risk, well-informed class (n=564)	76 (13.48; 10.90-16.54)	4.890 (3.224-7.418)	<.001	53 (9.40; 7.26-12.09)	3.035 (1.930-4.771)	<.001
Moderate-risk, poorly informed class (n=565)	13 (2.30; 1.35-3.90)	0.891 (0.463-1.713)	.73	6 (1.06; 0.49-2.30)	0.385 (0.158-0.939)	.04
High-risk, poorly informed class (n=952)	129 (13.55; 11.52-15.87)	5.419 (3.690-7.959)	<.001	61 (6.41; 5.02-8.15)	2.574 (1.666-3.978)	<.001

a Not applicable.

b The “high-risk, well-informed” class was the reference group.

In contrast, the moderate-risk, poorly informed group showed no significant difference in PrEP use (2.30%; OR 0.891, 95% CI 0.463‐1.713; *P*=.729) and had significantly lower PEP uptake (1.06%; OR 0.385, 95% CI 0.158‐0.939; *P*=.036) compared to the reference group.

## Discussion

### Principal Findings

This study identified 4 latent subgroups among men who have sex with men in Hangzhou based on sexual behaviors and prevention-related awareness (low-risk, well-informed; high-risk, well-informed; moderate-risk, poorly informed; and high-risk, poorly informed groups). PrEP and PEP use were primarily concentrated among the 2 high-risk groups, whereas the moderate-risk but poorly informed group exhibited particularly low uptake (PrEP: 2.30%; PEP: 1.06%), indicating a substantial mismatch between behavioral exposure and coverage of HIV prevention services.

### Determinants of Behavioral-Cognitive Profiles

Sociodemographic factors play a pivotal structural role in differentiating men who have sex with men subpopulations according to their HIV risk profiles. Education and income emerged as the most consistent predictors among the high-risk and moderate-risk but poorly informed groups. Lower educational attainment—particularly junior high school and vocational training—was significantly associated with membership in the high-risk, poorly informed group, while individuals with unstable residence and nonlocal household registration were more likely to fall into the moderate-risk, poorly informed group. These findings align with existing literature indicating that lower socioeconomic status is strongly associated with reduced access to HIV-related knowledge, rather than knowledge-use behaviors. For example, research from Brazil and China suggests that men who have sex with men with lower income and education levels tend to have lower HIV knowledge scores and weaker risk perception [35,36]. This phenomenon may be explained by structural factors such as informational exclusion, institutional discrimination, and unequal access to community-based education programs. Studies from both high- and middle-income countries also indicate that health communication tends to favor those with higher educational attainment, reinforcing knowledge gaps within socioeconomically marginalized men who have sex with men subgroups [37,38]. These findings highlight the need to design differentiated health promotion strategies that address the cognitive disadvantage created by broader structural inequities.

### Structural Inequalities and Implications for HIV Prevention

Both high-risk subgroups—irrespective of their cognition of HIV preventive measures—exhibited significantly elevated rates of PrEP and PEP use. This aligns with well-documented associations between high-risk sexual behaviors and the uptake of biomedical prevention tools observed among men who have sex with men globally, including in China [39,40]. Notably, PrEP usage among the high-risk, poorly informed group was comparable to that of their well-informed counterparts, despite markedly lower levels of HIV-related knowledge. This pattern suggests that PrEP uptake in this subgroup may be less influenced by comprehensive prevention literacy and more reflective of reactive, event-driven decision-making—for example, seeking PEP following perceived exposure, or initiating PrEP due to a partner’s HIV status or prior testing experiences. Similar behavioral dynamics have been reported in other men who have sex with men populations, where PrEP use is often shaped more by perceived vulnerability and episodic risk than by an in-depth understanding of biomedical interventions. For instance, a study in Belgium found that men who have sex with men modulated PrEP adherence based on fluctuating risk exposure, indicating a largely reactive usage model [41]. Likewise, research in India reported that men who have sex with men with higher perceived HIV risk were more willing to use PrEP, regardless of their actual knowledge of the intervention [42]. However, the rate of PEP use in the high-risk, poorly informed group (6.41%) was substantially lower than in the high-risk, well-informed group (9.40%). This discrepancy likely reflects the time-sensitive nature of PEP initiation—typically within 72 hours of exposure—which demands a higher degree of health awareness. The comparatively lower uptake in the low-literacy group underscores the urgent need for targeted health education to bridge this critical knowledge-to-action gap.

Interestingly, no significant difference in PrEP or PEP uptake was observed between the high-risk, well-informed and high-risk, poorly informed groups, challenging the assumption that cognition of HIV prevention measures alone directly translates into preventive behavior. Several factors may account for this disconnect. First, both groups may encounter similar structural barriers, such as limited access, financial constraints, and stigma [43-45], that hinder service use regardless of knowledge levels. Second, general awareness of HIV prevention may not equate to a heightened perception of personal risk, thereby weakening the motivation to initiate PrEP or PEP [46,47]. Third, the knowledge indicators used in the LCA may capture general informational exposure rather than specific, service-oriented literacy or navigational capacity. Fourth, men who have sex with men populations often demonstrate event-driven, reactive patterns of biomedical prevention use, such as initiating PEP following perceived exposure, rather than sustained, proactive engagement [48]. Finally, social norms and peer influences may exert a stronger effect than individual knowledge [43,45,49], especially in the first context.

The moderate-risk, poorly informed group emerged as a notably underserved population in this study. Despite reporting non-negligible behavioral risks—such as a 24.1% prevalence of multiple concurrent sexual partnerships—this group exhibited the lowest uptake of PrEP (2.30%) and PEP (1.06%). Strikingly, 99.8% of individuals in this class lacked adequate knowledge of PrEP or PEP (Table 3), revealing a profound gap in biomedical prevention coverage for what may be termed an “invisible risk group.” This misalignment between behavioral vulnerability and prevention engagement echoes findings from prior research in China. For instance, Wang et al [9] reported rising PEP usage in Shenzhen—from 3.92% in 2018 to 10.29% in 2020—yet noted that such uptake remained concentrated among high-risk populations, with limited reach among moderately at-risk individuals with low prevention literacy [50]. Importantly, the disconnect between moderate-risk individuals and biomedical prevention tools reflects not only gaps in personal awareness but also deeper structural and informational asymmetries. As Lei [51] highlights in a qualitative study, knowledge dissemination about PrEP and PEP within men who have sex with men communities in China is highly networked and stratified. Information circulates primarily through specific digital platforms and community-based networks, often excluding individuals from rural backgrounds, those with limited education, or recent migrants [51]. These mechanisms reinforce their “invisibility” in existing prevention infrastructures, rendering them unable to access timely information or interventions despite engaging in risk behaviors. This phenomenon is supported by quantitative evidence: studies in China have shown that low health literacy, rural or nonlocal household registration, recent migration, and limited access to public sector resources are independently associated with restricted access to HIV prevention services [52-54]. Traditional intervention strategies, which often prioritize those at the highest observable risk, may inadvertently neglect populations with moderate but sustained behavioral risk. For this structurally disconnected group, a multilevel intervention framework is urgently needed. This includes peer-led community education, embedding PrEP or PEP counseling into routine HIV testing, and implementing culturally responsive outreach for rural and migrant populations [52,55]. Enhancing both institutional design and the communication ecology will be essential for achieving equitable, targeted service delivery across diverse men who have sex with men subpopulations.

### Limitations

This study has several limitations. First, its cross-sectional design limits the ability to infer causal relationships between HIV risk patterns and PrEP or PEP uptake. Second, self-reported data may be subject to recall bias or social desirability bias, potentially affecting the accuracy of behavioral and prevention-related indicators. Third, participant recruitment was conducted exclusively online through a NGO’s digital platforms, which may have excluded men who have sex with men who are less digitally connected or more structurally marginalized. Fourth, PrEP and PEP usage were measured as binary variables, without capturing duration, adherence, or regimen type, thereby restricting the interpretability of biomedical prevention engagement. Finally, this study did not collect or report participants’ HIV serological results, and thus the HIV status of respondents could not be objectively verified, which may introduce uncertainty regarding the actual infection status. Despite these limitations, the identification of distinct behavioral-knowledge typologies offers meaningful insights for refining HIV prevention strategies.

### Conclusions

This study identified that the 4 distinct subgroups of HIV-related risk profiles among men who have sex with men and PrEP or PEP use were concentrated among high-risk individuals. By integrating behavioral and prevention-cognition indicators through LCA, the study revealed an underserved subgroup, men who have sex with men with moderate behavioral risk but very low prevention literacy, which traditional behavior–only frameworks fail to detect. These findings demonstrate that knowledge alone is insufficient to drive PrEP or PEP uptake and highlight the need for differentiated, equity-focused strategies that pair education with improved access and supportive service environments. Targeted community-based education, embedding PrEP or PEP counseling into low-threshold HIV testing, and outreach to men who have sex with men with lower socioeconomic status emerge as practical priorities to close prevention gaps. By offering a multidimensional, person-centered perspective, this study contributes novel evidence to inform real-world HIV prevention planning in China and other middle-income settings facing similar structural challenges.

## Supplementary material

10.2196/81405 Checklist 1 STROBE checklist.

## Abbreviations

aBIC: adjusted Bayesian information criterion
AIC: Akaike information criterion
ALCPP: average latent class posterior probability
BIC: Bayesian information criterion
BLRT: bootstrapped likelihood ratio test
LCA: latent class analysis
NGO: nongovernmental organization
PEP: postexposure prophylaxis
PrEP: pre-exposure prophylaxis
SPSS: Statistical Package for Social Sciences
STI: sexually transmitted infection
STROBE: Strengthening the Reporting of Observational Studies in Epidemiology
VLMR–LRT: Vuong-Lo-Mendell-Rubin likelihood ratio test
WHO: World Health Organization

## References

[1] (2024). Global HIV & AIDS statistics—fact sheet. Joint United Nations Programme on HIV/AIDS.

[2] (2023). Understanding fast-track: accelerating action to end the AIDS epidemic by 2030. Joint United Nations Programme on HIV/AIDS.

[3] Korenromp EL, Sabin K, Stover J (2024). New HIV infections among key populations and their partners in 2010 and 2022, by world region: a multisources estimation. J Acquir Immune Defic Syndr.

[4] Editorial Office of Chinese Journal of AIDS & STD (2025). National AIDS and sexually transmitted disease epidemic in December 2024. Chin J AIDS STD.

[5] Yin Y, Liu Y, Zhu J (2019). The prevalence, temporal trends, and geographical distribution of HIV-1 subtypes among men who have sex with men in China: a systematic review and meta-analysis. Epidemiol Infect.

[6] Ma Y, Dou Z, Guo W (2018). The human immunodeficiency virus care continuum in China: 1985–2015. Clin Infect Dis.

[7] (2021). Consolidated guidelines on HIV prevention, testing, treatment, service delivery and monitoring. World Health Organization.

[8] Acquired Immunodeficiency Syndrome Professional Group, Society of Infectious Diseases, Chinese Medical Association; Chinese Center for Disease Control and Prevention (2024). Chinese guidelines for the diagnosis and treatment of human immunodeficiency virus infection/acquired immunodeficiency syndrome (2024 edition). Chin Med J.

[9] Wang H, Tang W, Shang H (2022). Expansion of PrEP and PEP services in China. Lancet HIV.

[10] Liu S, Yu F, Xue H (2022). Factors influencing awareness and use of HIV prophylaxis medications among men who have sex with men in seven cities. China AIDS & STD.

[11] Jiang Z, Wang Q, Liang J (2024). Exploration of PrEP/PEP service delivery model in China: a pilot in eastern, central and western region. Glob Health Med.

[12] Liu Y, Liu X, Wei S (2024). Identifying patterns of sexual behaviors and PrEP uptake characteristics among MSM who were eligible for PrEP: a national cross-section study. J Virus Erad.

[13] Baggaley RF, White RG, Boily MC (2010). HIV transmission risk through anal intercourse: systematic review, meta-analysis and implications for HIV prevention. Int J Epidemiol.

[14] Okafor CN, Gorbach PM, Ragsdale A, Quinn B, Shoptaw S (2017). Correlates of preexposure prophylaxis (PrEP) use among men who have sex with men (MSM) in Los Angeles, California. J Urban Health.

[15] Guan Y, Qi T, Liao Q (2023). Multi-dimensional mismatch and barriers for promoting PrEP among men who have sex with men in China: a cross sectional survey from the Demand-side. AIDS Res Ther.

[16] de Sousa AFL, Lima SVMA, Ribeiro CJN (2023). Adherence to pre-exposure prophylaxis (PrEP) among men who have sex with men (MSM) in Portuguese-speaking countries. Int J Environ Res Public Health.

[17] He Y, Chu X, Li N (2025). Meta-analysis of factors influencing willingness to accept HIV post-exposure prophylaxis among Chinese men who have sex with men. Mod Prev Med.

[18] Kulie P, Castel AD, Zheng Z (2020). Targeted screening for HIV pre-exposure prophylaxis eligibility in two emergency departments in Washington, DC. AIDS Patient Care STDS.

[19] Ma Q, Jiang T, Chen W (2025). Awareness of sexual partner’s HIV status among men who have sex with men in China: cross-sectional survey study. JMIR Public Health Surveill.

[20] Xu JF, Wang PC, Cheng F (2020). Health related behaviors among HIV-infected people who are successfully linked to care: an institutional-based cross-sectional study. Infect Dis Poverty.

[21] Mgbako O, Park SH, Mayer KH (2019). Transactional sex and preferences for pre-exposure prophylaxis (PrEP) administration modalities among men who have sex with men (MSM). J Sex Res.

[22] Wang Z, Mo PKH, Ip M, Fang Y, Lau JTF (2020). Uptake and willingness to use PrEP among Chinese gay, bisexual and other men who have sex with men with experience of sexualized drug use in the past year. BMC Infect Dis.

[23] Lin H, Li JH, Yang X (2023). Discrepancy between behavioral-indicated and perceived candidacy for HIV pre-exposure prophylaxis among men who have sex with men in Chengdu, China. Beijing Da Xue Xue Bao Yi Xue Ban.

[24] Tang V, Montemayor BN, Owens C (2025). Factors influencing oral pre-, post-, and doxycycline post-exposure prophylaxis uptake among substance-using men who have sex with men in the rural southern US. AIDS Patient Care STDS.

[25] Dai S, Zhang D, Zhang J (2024). Survey on the current status of HIV pre- and post-exposure prophylaxis awareness and service usage among men who have sex with men (MSM). Chin J Dis Control Prev.

[26] von Elm E, Altman DG, Egger M (2007). The Strengthening the Reporting of Observational Studies in Epidemiology (STROBE) statement: guidelines for reporting observational studies. PLoS Med.

[27] Zhang X, Qi SZ, Du FZ (2022). Awareness and willingness to accept syphilis chemoprophylaxis among men who have sex with men from three cities in China: a cross-sectional study. BMC Public Health.

[28] Chen N (2024). A study on pre- and post-exposure prophylaxis medication for HIV among men who have sex with men based on social network analysis [Master’s Thesis].

[29] Liu Z (2023). Study on the Current Status and Related Factors Inself-Protective Behaviors for the Prevention of HIV Infection Among Men Who Have Sex With Men [Master’s Thesis].

[30] Hoijtink H (2001). Confirmatory latent class analysis: model selection using Bayes factors and (pseudo) likelihood ratio statistics. Multivariate Behav Res.

[31] Masyn KE (2013). The Oxford Handbook of Quantitative Methods in Psychology: Statistical Analysis.

[32] Asparouhov T, Muthén B (2014). Auxiliary variables in mixture modeling: three-step approaches using Mplus. Struct Equ Modeling.

[33] Nylund KL, Asparouhov T, Muthén BO (2007). Deciding on the number of classes in latent class analysis and growth mixture modeling: a Monte Carlo simulation study. Struct Equ Modeling.

[34] Nylund-Gibson K, Choi AY (2018). Ten frequently asked questions about latent class analysis. Transl Issues Psychol Sci.

[35] Guimarães MDC, Magno L, Ceccato MGB (2019). HIV/AIDS knowledge among MSM in Brazil: a challenge for public policies. Rev Bras Epidemiol.

[36] Fan W, Yin L, Qian HZ (2014). HIV risk perception among HIV negative or status-unknown men who have sex with men in China. Biomed Res Int.

[37] Kiviniemi MT, Orom H, Waters EA, McKillip M, Hay JL (2018). Education-based disparities in knowledge of novel health risks: the case of knowledge gaps in HIV risk perceptions. Br J Health Psychol.

[38] Philbin MM, Hirsch JS, Wilson PA, Ly AT, Giang LM, Parker RG (2018). Structural barriers to HIV prevention among men who have sex with men (MSM) in Vietnam: diversity, stigma, and healthcare access. PLoS ONE.

[39] Jin J, Sun R, Mu T (2022). Awareness and use of post-exposure prophylaxis for HIV prevention among men who have sex with men: a systematic review and meta-analysis. Front Med (Lausanne).

[40] Du J, Wang S, Zhang H (2025). Pre-exposure Prophylaxis (PrEP) awareness and engagement among MSM at high risk of HIV infection in China: a multi-city cross-sectional survey. AIDS Behav.

[41] Vuylsteke B, Reyniers T, De Baetselier I (2019). Daily and event-driven pre-exposure prophylaxis for men who have sex with men in Belgium: results of a prospective cohort measuring adherence, sexual behaviour and STI incidence. J Int AIDS Soc.

[42] Sun Z, Gu Q, Dai Y (2022). Increasing awareness of HIV pre-exposure prophylaxis (PrEP) and willingness to use HIV PrEP among men who have sex with men: a systematic review and meta-analysis of global data. J Int AIDS Soc.

[43] Sun Y, Lu H, Ye J, Li D, Li G (2023). Awareness and use of HIV pre-exposure prophylaxis and factors associated with awareness among MSM in Beijing, China. Sci Rep.

[44] Mayer KH, Agwu A, Malebranche D (2020). Barriers to the wider use of pre-exposure prophylaxis in the United States: a narrative review. Adv Ther.

[45] Guo JH, Zhang G, Qin QQ, Chen HJ, Wang L, Lyu F (2022). Progress in research of knowledge, attitude and practice of pre-exposure prophylaxis in men who have sex with men and its influencing factors. Zhonghua Liu Xing Bing Xue Za Zhi.

[46] Blumenthal J, Jain S, Mulvihill E (2019). Perceived versus calculated HIV risk: implications for pre-exposure prophylaxis uptake in a randomized trial of men who have sex with men. J Acquir Immune Defic Syndr.

[47] Kota KK, Gelaude D, Carnes N (2024). Low self-perceived need for PrEP and behavioral indications of MSM who recently refused daily PrEP: a mixed methods study in three US cities. AIDS Behav.

[48] Beymer MR, Bolan RK, Flynn RP (2014). Uptake and repeat use of postexposure prophylaxis in a community-based clinic in Los Angeles, California. AIDS Res Hum Retroviruses.

[49] Quinn KG, Christenson E, Spector A, Amirkhanian Y, Kelly JA (2020). The influence of peers on PrEP perceptions and use among young black gay, bisexual, and other men who have sex with men: a qualitative examination. Arch Sex Behav.

[50] Wang Y, Liu S, Zhang Y (2022). Use of HIV post-exposure prophylaxis among men who have sex with men in Shenzhen, China: a serial cross-sectional study. AIDS Behav.

[51] Lei Z (2023). Accessibility, utilization and governmentality: a qualitative research on prep in MSM community [Master’s Thesis].

[52] Chen W, Chen L, He L, Chai C (2024). Urban-rural disparity in risky sexual behavior, HIV knowledge, and healthy practices among men who have sex with men: a cross-sectional study in Southeast China. PLoS ONE.

[53] Wu J, Wu H, Li P, Lu C (2016). HIV/STIs risks between migrant MSM and local MSM: a cross-sectional comparison study in China. PeerJ.

[54] Jaiswal J, Griffin M, Singer SN (2018). Structural barriers to pre-exposure prophylaxis use among young sexual minority men: the P18 cohort study. Curr HIV Res.

[55] Li C, Xiong Y, Muessig KE (2022). Community-engaged mHealth intervention to increase uptake of HIV pre-exposure prophylaxis (PrEP) among gay, bisexual and other men who have sex with men in China: study protocol for a pilot randomised controlled trial. BMJ Open.

